# Temperature-Adaptive Excitation Technology for Acoustic Logging Monopole Transducers

**DOI:** 10.3390/s26041089

**Published:** 2026-02-07

**Authors:** Kai Zhang, Xinyan Wang, Baohai Tan, Yuanda Su

**Affiliations:** 1 State Key Laboratory of Deep Oil and Gas, China University of Petroleum (East China), Qingdao 266555, China; tanbaohai@upc.edu.cn (B.T.); suyuanda@upc.edu.cn (Y.S.); 2 School of Geosciences and Technology, China University of Petroleum (East China), Qingdao 266555, China; z24010048@s.upc.edu.cn

**Keywords:** sonic logging, monopole transducer, excitation circuit, adaptive adjustment, impedance matching, temperature sensitivity

## Abstract

Acoustic logging tools, deployed thousands of meters underground to detect geological structures and evaluate reservoir fluids, are essential for oil and gas exploration and development. These tools generate acoustic signals through piezoelectric ceramic transducers. The material properties of piezoelectric ceramics are significantly affected by the high-temperature downhole environment, leading to a failure in impedance matching between the transducer and its excitation circuit. This results in a substantial degradation of the tool’s performance. This paper experimentally obtains the electrical parameters and excitation energy of commonly used monopole transducers at different temperatures. Based on this data, the optimal matching inductance values at various temperatures are calculated. A temperature-adaptive transducer excitation circuit is then designed and implemented. This circuit can adjust the excitation frequency according to the measured temperature to compensate for resonant frequency drift and select the optimal inductor tap via a programmable multiplexer. Experimental results demonstrate that this circuit significantly enhances the transducer’s excitation energy at high temperatures. This technology is expected to markedly improve the operational stability of acoustic logging tools and facilitate the exploration and development of deep and ultra-deep oil and gas resources.

## 1. Introduction

Acoustic logging is a core technology for exploring underground oil and gas resources [1,2,3,4]. This method interprets formation structures [5,6] and pore characteristics at depths of several thousand meters by analyzing the propagation characteristics of acoustic waves through rock layers [7]. Currently, the acoustic sources used in this technology are predominantly piezoelectric ceramic transducers [8]. Evolution in the materials, structure, and performance of these transducers has driven the development of acoustic logging technology. Presently, transducers can be broadly categorized into monopole [9], dipole [10], and multipole types used in logging-while-drilling (LWD) applications [11]. Among these, the monopole transducer employs a cylindrical structure to generate compressional waves, with a resonant frequency typically around 15 kHz to 20 kHz [12], representing the most fundamental and mature technology in this field. Owing to its structural simplicity, early adoption, and high robustness, the monopole configuration represents the earliest developed and most mature acoustic source in borehole logging, and it remains the most widely deployed transmitter in current wireline and logging-while-drilling tools.

Extensive studies have been conducted on the effects of structure, materials, and mounting methods on the excitation bandwidth and power of monopole transducers [13,14,15,16,17]. However, systematic research into how their performance varies under high-temperature conditions remains scarce. In fact, acoustic logging tools often operate in high-temperature environments (200 °C) [18,19], where significant shifts in the transducer’s resonant frequency occur, along with notable changes in key parameters such as piezoelectric constants, dielectric constants, and electromechanical coupling coefficients.

The excitation circuit of the transducer typically employs a step-up transformer to generate high-voltage electrical signals in the range of several kilovolts. In electronic systems, the excitation circuit and its transformer can be regarded as inductive components, while the transducer behaves as a capacitive component. Impedance mismatch between them can lead to reactive power, reduced excitation energy, and even cause component heating or damage [20,21]. Therefore, additional inductors or capacitors are typically added to the excitation circuit to achieve impedance matching. Nevertheless, the material parameters of the transducer vary significantly with temperature, leading to drastic changes in its impedance characteristics [22,23,24]. Under such conditions, the matching circuit designed under room temperature becomes ineffective, leading to degraded stability of the acoustic source during deep formation measurements.

To address the aforementioned issues, this paper investigates the high-temperature characteristics and compensation techniques for monopole transducers. Temperature sensitivity tests were conducted on the electrical parameters of commonly used monopole transducers in acoustic logging. Building on the test results, a temperature-adaptive transducer excitation circuit was designed. This circuit dynamically adjusts both excitation frequency and matching inductance in response to ambient temperature variations, thereby ensuring consistent transducer performance across different formation depths.

## 2. Experimental Testing of High-Temperature Performance of Monopole Transducers

Monopole transducers commonly used in acoustic logging typically employ PZT-5G piezoelectric ceramic [13,25], which has a Curie temperature of 380 °C and offers advantages such as excellent temperature resistance and high electromechanical conversion efficiency. However, despite these advantages, PZT-5G is not widely adopted in the industry, and experimental data on the temperature-dependent variations of its key properties (such as dielectric constants, piezoelectric constants, and elastic moduli) remain unavailable. This makes it impossible to study them through theoretical calculations or numerical simulations. To address this gap, this study acquired five monopole cylindrical transducers [26] of identical structure (with an inner diameter of 60 mm, outer diameter of 70 mm, and height of 76 mm). Experimental methods were employed to determine the variation patterns of their key parameters with temperature, and subsequent research was conducted based on these findings.

### 2.1. Temperature Sensitivity Test of Transducer Electrical Parameters

**Figure 1:**
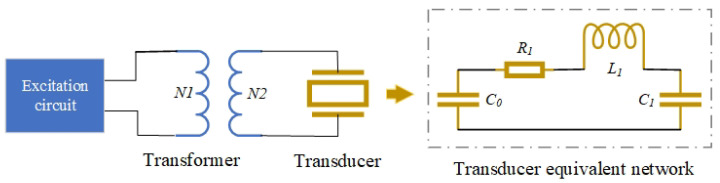
Transducer Electrical Connection Diagram and Its Equivalent Network Model.

Testing the electrical parameters of a transducer requires reference to its equivalent circuit model [27,28]. A commonly used lumped-parameter equivalent model is shown in Figure 1, where $C_{0}$ represents the static capacitance. This parameter plays a dominant role when the excitation voltage is DC or low-frequency, serving as a key factor in the impedance matching between the transducer and the excitation circuit, and directly influencing the excitation bandwidth of the transducer. $R_{1}$, $L_{1}$, and $C_{1}$ represent the dynamic resistance, dynamic inductance, and dynamic capacitance, respectively. These parameters become dominant when the excitation voltage is high-frequency (particularly near the resonant frequency), fundamentally determining key performance indicators of the transducer, such as the resonant frequency and transmitting voltage response level.

**Figure 2:**
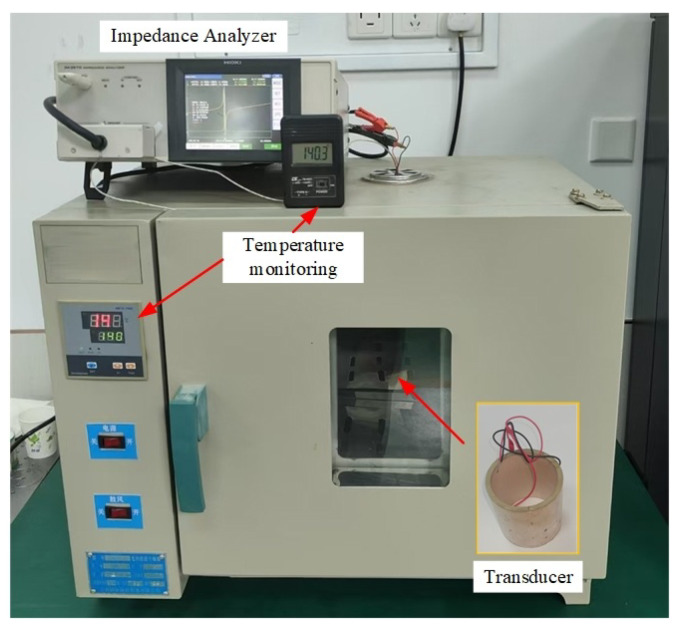
On-site Testing Diagram of Transducer Electrical Parameters.

The transducer was placed in a high-temperature oven for heating, and its resonant frequency and equivalent network parameters were measured at different temperatures using a high-precision impedance analyzer (HIOKI IM3570) (Figure 2). The furnace temperature was regulated by a PID temperature controller with a control accuracy of ±0.2 °C. The temperature was monitored using a calibrated sensor with an accuracy of ±0.15 °C, placed in close proximity to the transducer. At each temperature setpoint, the transducer was held under isothermal conditions for 10 min prior to measurement to ensure thermal equilibrium. During each impedance measurement, the temperature remained stable without observable drift. Given the relatively small dimensions of the transducer and the applied soaking time, any internal temperature gradient within the sample is considered negligible.

**Figure 3:**
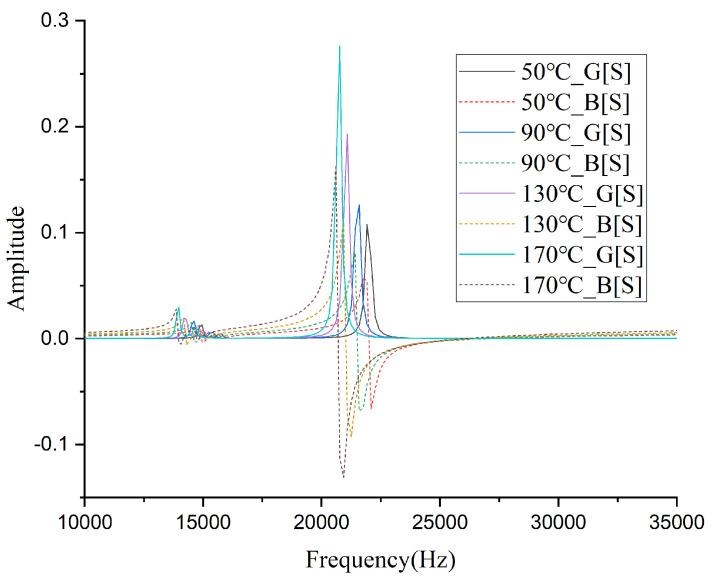
Conductance (G) and susceptance (B) spectra of the monopole transducer measured at representative temperatures.

Statistical analysis demonstrated no significant differences among the five samples, so the average value of the measurement results was taken. The measured values of various parameters with temperature are shown in Table 1. Figure 3 presents the conductance (G) and susceptance (B) spectra of the monopole transducer measured at several representative temperatures. As the temperature increases, the resonant frequency of the transducer decreases, with a 10% reduction (9.96% precisely) at 210 °C compared to room temperature. The static capacitance and dynamic capacitance at 210 °C increase by approximately 2.4 times and 2.7 times, respectively, while the dynamic resistance and dynamic inductance decrease to about 23% and 26% of their room temperature values. These changes are attributed to the variations in the relative permittivity, piezoelectric constant, and elastic constants of the piezoelectric ceramic material induced by high temperatures [29].

### 2.2. Transducer Excitation Energy Temperature Sensitivity Test

While the equivalent network model of the transducer indicates significant performance variations under high temperatures, the actual energy excited by the transducer depends on multiple additional parameters. Furthermore, real-world engineering conditions are far more complex than theoretical models. Therefore, direct testing of the energy excited by the transducer is necessary. Conventional high-temperature testing often employs a thermal shield between the transmitting and receiving transducers, allowing the transmitter to heat while the receiver remains at a stable temperature [12]. However, achieving an optimal trade-off between acoustic transparency and thermal insulation is difficult, and shield performance is highly temperature dependent, which can introduce measurement errors.

**Figure 4:**
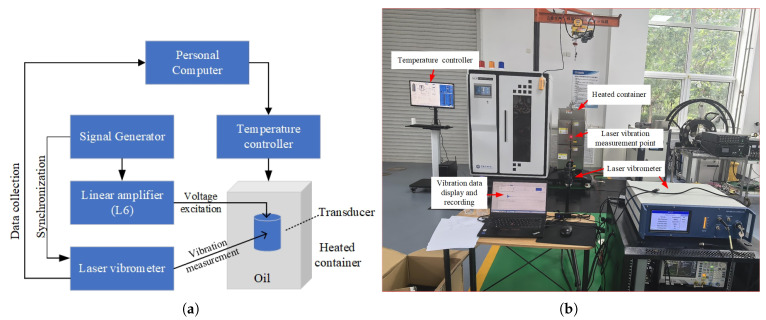
Connection diagram (**a**) and physical setup (**b**) of the experimental apparatus for testing the transducer’s excitation energy.

To overcome these limitations, we used a laser vibrometer to measure the container shell’s vibration as an indirect indicator of transmitted energy [30], without the need for direct contact. Figure 4 shows the connection diagram of the experimental setup. The workflow is as follows: (1) A temperature controller heats the oil-filled container; (2) A signal generator continuously produces three cycles of a low-voltage sine wave per second (frequency: 20 kHz), which is amplified to 2000 V by a linear power amplifier; (3) The high-voltage signal is applied to the monopole transducer to generate acoustic waves, which induce mechanical vibrations in the container’s outer wall; (4) The signal generator outputs a synchronization signal to the laser vibrometer for synchronous acquisition of vibration data.

A custom high-temperature chamber was used with temperature controlled to within ±0.2 °C. Vibration measurements were acquired using a Doppler laser vibrometer (SOPTOP LV-SC400-P) at a sampling rate of 50 MHz, enabling high-resolution capture of transducer motion. The excitation was implemented on custom boards: key components include a 16-bit digital-to-analog converter (DAC, Analog Devices AD5600) driving the transmitter, and a 16-bit successive-approximation ADC (Analog Devices AD7981) for receiver acquisition. The laser vibrometer was calibrated using a standard reference vibration source prior to testing; the combined uncertainty of temperature control and vibration measurement is estimated at ±5% across the tested range.

To validate the use of vibration amplitude as a proxy for transmitted acoustic energy under high-temperature conditions, a dedicated experiment was conducted in an anechoic water tank. The same excitation circuit and transducer were used, and controlled excitation voltages were applied while hydrophone recordings of the radiated acoustic signals were acquired. A linear relationship between excitation voltage and received acoustic amplitude was established. The same excitation voltage set was applied in the high-temperature chamber with vibration amplitude measured by laser vibrometry. A comparison of the two sets of amplitude vs. voltage curves yielded a high linear correlation ($R^{2}>0.95$), confirming that vibration amplitude measured in the chamber reliably reflects transmitted acoustic energy.

Although parameters such as the oil and the container’s outer wall also vary with temperature, the structural design and material selection of this setup are based on actual wellbore conditions (steel casing and drilling fluid), making the experimental results more representative of real logging environments.

**Figure 5:**
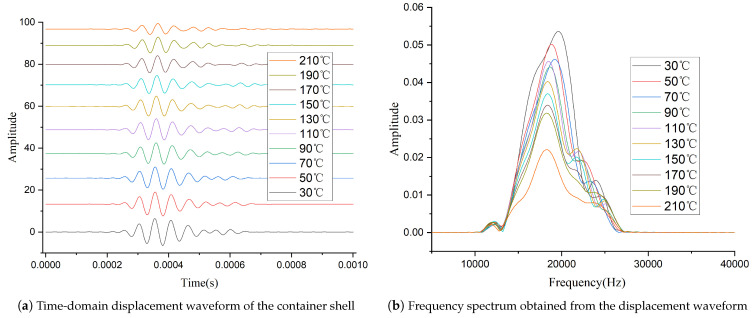
Vibration responses of the container shell measured by the laser vibrometer at different temperatures: (**a**) time-domain displacement waveforms; (**b**) corresponding frequency spectra obtained by Fourier transformation.

Figure 5 shows the vibration responses of the container shell at different temperatures, measured by the laser vibrometer and processed with a 10–30 kHz bandpass filter, where Figure 5a presents the time-domain displacement waveforms of the container shell vibration, and Figure 5b shows the corresponding frequency spectra obtained by Fourier transformation of the displacement signals. Analysis of these data shows that the transducer’s excitation energy decreases with increasing temperature, with a notable acceleration around 200 °C—close to half the material’s Curie temperature [31]. In addition, the center frequency of the excitation waveform tends to decrease with increasing temperature, which is consistent with the change in the transducer’s equivalent network parameters with temperature. The temperature-dependent variation in excitation energy observed in Figure 5 is consistent with the electrical parameter changes summarized in Table 1. As the transducer temperature increases, its dynamic resistance decreases significantly (Table 1), which leads to a pronounced increase in excitation current. Because the available output power of the excitation circuit is limited, the excitation voltage exhibits a moderate decrease with increasing temperature. Under fixed impedance-matching conditions, the temperature-induced impedance variation introduces an increasing phase difference between voltage and current, resulting in additional reactive power and a reduction in the effective energy delivered to the transducer. These electrical effects jointly explain the decreasing vibration amplitude and spectral magnitude observed in Figure 5.

The two experiments above demonstrate that the transducer’s resonant frequency decreases slightly with increasing temperature, and the excitation energy decreases significantly. This serves as the primary reason for the performance degradation of conventional acoustic logging tools when operating in deep and ultra-deep downhole environments.

## 3. High-Temperature Adaptive Transducer Excitation Circuit Design

### 3.1. Calculation of Matching Inductance Between Transducer and Excitation Circuit

The impedance matching of the transducer is based on the equivalent network model shown in Figure 1. When the transducer operates at its resonant frequency, the dynamic branch components $C_{1}$ and $L_{1}$ undergo series resonance, i.e., $\omega L_{1}=\frac{1}{\omega C_{1}}$. At this point, the dynamic branch is equivalent to a pure resistor $R_{1}$, and its complex impedance is $Z_{R1}=R_{1}$. Meanwhile, the complex impedance formula of the static branch $C_{0}$ is:

(1) $$Z_{C0}=\frac{1}{j\omega C_{0}}=-\frac{j}{\omega C_{0}}.$$

The transducer is thus equivalent to the parallel connection of $C_{0}$ and $R_{1}$. According to the complex impedance formula of the parallel circuit, the total impedance Z is:

(2) $$Z_{i}=\frac{Z_{R1}\cdot Z_{C0}}{Z_{R1}+Z_{C0}}=\frac{R_{1}\cdot \left(-\frac{j}{\omega C_{0}}\right)}{R_{1}-\frac{j}{\omega C_{0}}}.$$

The above formula can be further deduced as:

(3) $$Z_{i}=R_{i}+jX_{i}=\frac{R_{1}}{1+\omega_{0}^{2}C_{0}^{2}R_{1}^{2}}-j\frac{\omega_{0}R_{1}^{2}C_{0}}{1+\omega_{0}^{2}R_{1}^{2}C_{0}^{2}}.$$

From the above derivation, it can be observed that the transducer exhibits capacitive characteristics in the electronic system, while the transformer presents inductive behavior due to its magnetic core winding. Among the various methods to achieve impedance matching between them, the most convenient and effective approach is to connect an inductor in series with the primary winding of the transformer [32].

According to the impedance transformation formula of an ideal transformer, if the secondary impedance (transducer side) is $Z_{2}$, the primary impedance (excitation source side) is $Z_{1}$, and the turns ratio is $n=n_{1}/n_{2}$, where $n_{1}$ is the number of primary winding turns and $n_{2}$ is the number of secondary winding turns, the relationship is given by $Z_{1}=n^{2}*Z_{2}$. To offset the capacitive reactance $X_{i}$ of the secondary and make the primary present a pure resistive characteristic, an inductor *L* needs to be connected in series with the primary to make the inductive reactance $\left(X_{L}=\omega L\right)$ equal to the capacitive reactance converted from the secondary to the primary $\left(|n^{2}X_{i}|\right)$. That is:

(4) $$\omega L=|n^{2}X_{i}|.$$

From Equation (3), it can be deduced:

(5) $$X_{i}=-\frac{\omega R_{1}^{2}C_{0}}{1+\omega^{2}C_{0}^{2}R_{1}^{2}}.$$

The formula for calculating the series inductance can be derived by combining Equations (4) and (5) as:

(6) $$L=n^{2}\cdot \frac{R_{1}^{2}C_{0}}{1+\omega^{2}C_{0}^{2}R_{1}^{2}}$$

**Figure 6:**
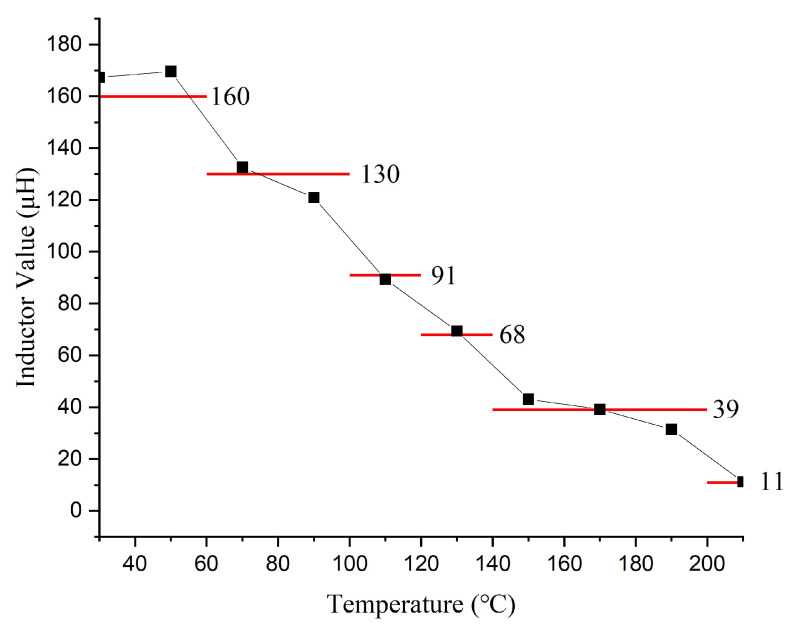
Calculated (black dots) and selected (red line) matching inductance values across temperatures.

Applying the data in Table 1 to Equation (6) yields the matching inductance values across temperatures (Figure 6), based on a fixed transformer turns ratio (*n*) of 1:10 and a leakage inductance of 25 μH. The results clearly indicate a significant decreasing trend in the required matching inductance as the temperature increases. Thereafter, to approximate the temperature-dependent inductance using a limited number of realizable values, a piecewise constant representation was employed over the temperature range of 30–210 °C.

Since the inductance exhibits a more rapid decrease at elevated temperatures, the temperature domain was divided into six non-uniform intervals, with coarser segmentation at lower temperatures and finer segmentation at higher temperatures to reduce the global approximation error under a fixed number of discrete levels.

Within each temperature interval, the representative inductance was determined as the arithmetic mean of the calculated inductance values in that interval, which is optimal in the least-squares sense and minimizes the root-mean-square error (RMSE) of a constant approximation. To ensure practical implementation, the representative values were further mapped to manufacturable inductance levels.

The six temperature intervals and their corresponding representative inductance values are summarized as follows: 30–60 °C (160 μH), 50–100 °C (130 μH), 100–120 °C (91 μH), 120–140 °C (68 μH), 140–200 °C (39 μH), and 200–210 °C (11 μH). The resulting approximation achieved an overall RMSE of approximately 3.77 μH before rounding and 5.61 μH after quantization, with the maximum absolute error remaining below 10 μH. This approach provides a balanced trade-off between approximation accuracy and manufacturing feasibility.

### 3.2. Design of Adaptive Excitation Electronic System

Based on the experimental and calculated results, the transducer excitation circuit requires two adjustments at high temperatures: first, adjusting the excitation signal frequency to track the resonant frequency shift, which is achieved by modifying the low-voltage control waveform in the main controller; second, adjusting the matching inductance to compensate for temperature-induced variations in the transducer’s electrical parameters. For the latter, a 6-tap inductor coil with values of 11, 39, 68, 91, 130, and 160 μH was designed, as indicated by the red line in Figure 6.

**Figure 7:**
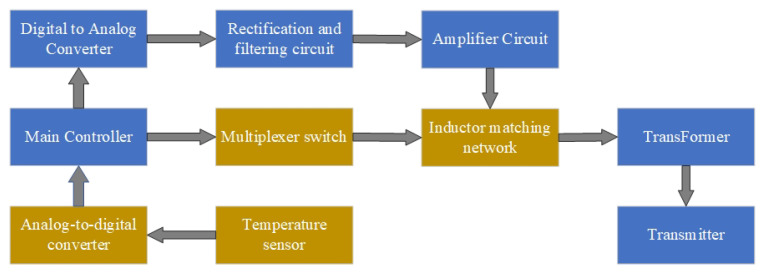
Block diagram of the temperature-adaptive excitation circuit for the monopole transducer.

The functional block diagram of the high-temperature adaptive acoustic logging transducer excitation circuit is shown in Figure 7. The components within the blue dashed box represent the traditional excitation circuit [33,34]. The main controller, implemented with an embedded Digital Signal Processor (DSP) [35], generates a low-voltage control waveform with specific frequency and amplitude. This waveform is converted into an analog signal by a Digital-to-Analog Converter (DAC), then undergoes filtering and amplification. After impedance matching, it is fed into a transformer for voltage step-up, ultimately driving the transducer.

To achieve adaptive impedance matching, the ambient temperature is collected, converted to digital signals by an analog-to-digital converter (ADC), and transmitted to the main controller. The main controller then performs a table lookup based on the temperature to determine the optimal matching inductor. A multiplexer switch (ADG408) is controlled to select the appropriate inductor values for optimal impedance matching. Each inductor tap corresponds to a discrete impedance-matching point pre-characterized over temperature. The selected inductors are connected in series with the transducer through the multiplexer, enabling stepwise adjustment of the effective matching inductance. The ADG408 offers low on-resistance and adequate isolation for the operating frequency range of the dipole transmitter, ensuring that the switching network introduces negligible additional loss or phase distortion. To further improve robustness near temperature interval boundaries, a temperature hysteresis margin is introduced in the inductance selection logic. Specifically, an additional transition band is defined around each temperature boundary, and the inductor tap is updated only when the measured temperature crosses this extended threshold. This hysteresis-based strategy prevents frequent switching when the temperature fluctuates near matching-value transitions and ensures stable inductance selection. Since inductance switching is only performed between excitation cycles, no transient artifacts are introduced during signal transmission.

## 4. Test of the Adaptive Adjustment Performance of the Excitation Circuit

**Figure 8:**
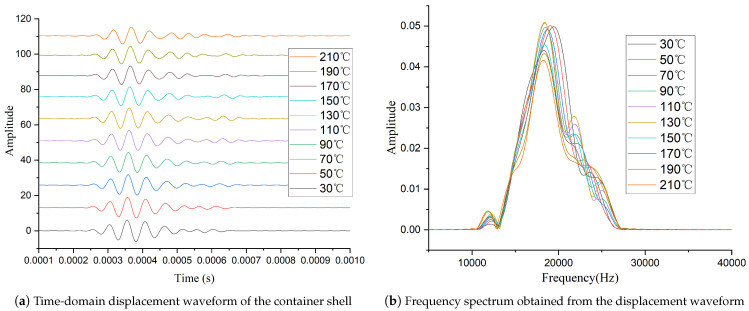
Time-domain vibration waveforms (**a**) and frequency spectrum waveforms (**b**) Excited by the Adaptive Excitation Circuit at Different Temperatures.

**Figure 9:**
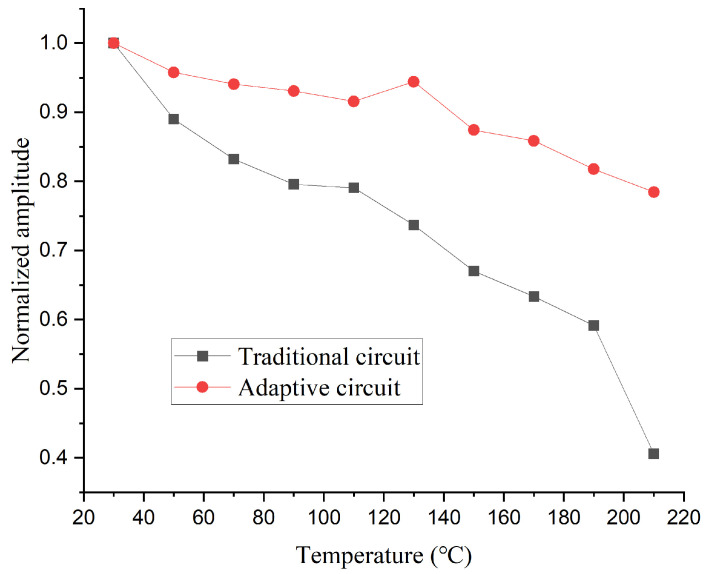
Normalized Peak-to-Peak Values of Signals Measured with the Two Circuits at Various Temperatures.

After completing the circuit design, we conducted tests under the same conditions using the setup shown in Figure 4. The time and frequency domain waveforms measured by the laser vibrometer are shown in Figure 8. Compared to Figure 5, it can be observed that although the amplitude of the excitation signal still decreases as temperature rises, the extent of this decline has been significantly reduced. Figure 9 shows the normalized peak-to-peak values of the signals measured with the two different circuits at various temperatures. The results demonstrate that the high-temperature adaptive adjustment circuit effectively maintains the stability of excitation energy across the tested temperature range.

To further quantify the effect of adaptive impedance matching, Table 2 summarizes the electrical parameters of the excitation circuit at representative temperatures. The excitation circuit operates approximately as a constant-power source, with an initial pulse width of 50 μH determined by the nominal transducer resonance frequency [33], while the preset excitation voltage was fixed at 3800 V.

As temperature increases, the transducer dynamic resistance decreases, resulting in a gradual rise in excitation current and a corresponding reduction in excitation voltage due to the limited output power. Under conventional excitation with fixed matching, temperature-induced impedance variations lead to a nonzero phase difference between voltage and current, introducing reactive power and reducing effective energy transfer, accompanied by a slight narrowing of the excitation pulse width.

In contrast, impedance-adaptive excitation actively compensates for temperature-dependent impedance changes. As shown in Table 2, this approach modifies the voltage–current phase behavior and maintains a higher excitation voltage at elevated temperatures, thereby improving real power transfer. It should be noted that the acoustic output is also influenced by temperature-dependent variations in resonant frequency and material properties; therefore, Table 2 primarily illustrates the electrical phase-regulation mechanism underlying the enhanced excitation stability.

## 5. Conclusions and Discussion

To address the issue of reduced excitation efficiency of acoustic logging monopole transducers in high-temperature downhole environments, this study experimentally measured the electrical parameters and excitation energy variations of the transducer at different temperatures. Based on the measurements, the impedance matching inductance values between the transducer and the excitation circuit at various temperatures were calculated. A tapped inductor with six selectable values was designed according to the computational results. Furthermore, a temperature-adaptive transducer excitation circuit was developed, which continuously monitors the ambient temperature. The circuit implements a dual adjustment strategy: it dynamically adjusts the excitation frequency to track shifts in resonant frequency, and employs a programmable multiplexer to select the appropriate inductance value for optimal impedance matching.

As the temperature rises, the resonant frequency of the transducer decreases. The equivalent network parameters of the transducer undergo significant changes: at 210 °C, the static capacitance and dynamic capacitance increase by approximately 2.4 times and 2.7 times, respectively, compared to their values at room temperature (30 °C), while the dynamic resistance and dynamic inductance decrease to about 23% and 26% of their room temperature values.

As the temperature rises, the transducer’s excitation energy decreases significantly, and the rate of decrease accelerates significantly near the half-Curie temperature. At this point, the waveform amplitude is approximately 40% of that at room temperature. This phenomenon is the primary direct cause of performance degradation in acoustic logging tools operating under high-temperature downhole conditions.

Based on the equivalent circuit model of the transducer, an inductor is connected in series with the primary winding of the transformer for impedance matching. The required impedance matching inductance values at different temperatures were calculated, leading to the design of a six-tap inductor coil with values of 11, 39, 68, 91, 130, and 160 μH respectively.

A temperature-adaptive transducer excitation circuit was designed to measure the ambient temperature in real time and dynamically adjust the transducer’s excitation frequency to track resonant frequency shifts. Simultaneously, the circuit employs a programmable multiplexer to select the optimal inductor tap for impedance matching.

High-temperature experimental results demonstrate that the adaptive excitation circuit significantly enhances the transducer’s excitation energy across different temperatures. At 210 °C, the excitation amplitude maintains approximately 82% of its room temperature value.

In addition, the electrical parameter comparison in Table 2 provides further evidence of the effectiveness of the adaptive excitation strategy. Under conventional excitation with fixed impedance matching, temperature-induced impedance variations introduce a voltage–current phase difference, increasing reactive power and reducing effective energy transfer. By contrast, impedance-adaptive excitation dynamically regulates the matching state with temperature, thereby adjusting the phase relationship and maintaining a higher excitation voltage at elevated temperatures. This electrical phase-regulation mechanism helps explain the improved excitation stability observed under high-temperature conditions.

After optimization by the adaptive excitation circuit, the consistency of the transducer’s excitation performance has been significantly improved. However, a notable performance gap still exists between high-temperature and room-temperature conditions. This discrepancy may be primarily attributed to increased dielectric losses under high temperatures and pre-stress relaxation caused by thermal expansion of the transducer.

To further improve performance consistency, adjusting the excitation voltage with temperature could be explored. However, as the dynamic resistance decreases at high temperatures, this approach would further increase power consumption. On the other hand, due to the increase in piezoelectric constants and the reduction in maximum safe strain, the safe excitation voltage for the transducer would also decrease significantly. Raising the excitation voltage might easily cause irreversible damage to the transducer, requiring comprehensive and in-depth research.

## Figures and Tables

**Table 1:** Variation of Electrical Parameters of Monopole Transducer with Temperature.

Temp. (°C)	Frequency		$C_{0}$		$R_{1}$		$L_{1}$		$C_{1}$	
	Val. (Hz)	Var. (%)	Val. (nF)	Var. (%)	Val. (Ω)	Var. (%)	Val. (mH)	Var. (%)	Val. (nF)	Var. (%)
30	20,135	0.00	23.74	0.00	28.59	0.00	4.42	0.00	12.81	0.00
50	19,924	−1.05	25.33	6.70	27.84	−2.62	4.17	−5.66	13.89	8.43
70	19,756	−1.88	28.09	18.32	23.76	−16.89	4.11	−7.01	14.31	11.71
90	19,422	−3.54	30.15	27.00	21.36	−25.29	3.39	−23.30	17.63	37.63
110	19,258	−4.36	31.83	34.08	16.32	−42.92	3.24	−26.70	19.08	48.95
130	19,094	−5.17	39.29	65.50	15.54	−45.65	2.66	−39.82	23.55	83.84
150	18,772	−6.77	44.12	85.85	12.45	−56.45	2.35	−46.83	27.59	115.38
170	18,612	−7.56	54.66	130.24	10.86	−62.01	1.98	−55.20	32.76	155.74
190	18,478	−8.23	66.75	181.17	9.21	−67.79	1.43	−67.65	38.42	199.92
210	18,130	−9.96	80.39	238.63	6.72	−76.50	1.17	−73.53	47.33	269.48

**Table 2:** Measured electrical parameters of the excitation circuit at representative temperatures.

Temp. (°C)	Excitation Mode	Voltage (V)	Pulse Width (μs)	Current Peak (A)	Phase (°)
30	Conventional excitation	3800	50.00	0.210	0.6
	Impedance-adaptive excitation	3800	50.00	0.210	0.0
50	Conventional excitation	3705	49.20	0.221	1.1
	Impedance-adaptive excitation	3740	49.40	0.221	6.8
70	Conventional excitation	3608	48.60	0.233	0.9
	Impedance-adaptive excitation	3695	48.90	0.234	12.5
90	Conventional excitation	3542	48.25	0.237	1.5
	Impedance-adaptive excitation	3649	48.60	0.237	18.4
110	Conventional excitation	3410	47.48	0.251	0.8
	Impedance-adaptive excitation	3559	47.93	0.252	24.7
130	Conventional excitation	3277	46.71	0.265	1.9
	Impedance-adaptive excitation	3470	47.26	0.266	31.6
150	Conventional excitation	3105	46.10	0.287	1.2
	Impedance-adaptive excitation	3288	46.65	0.289	38.9
170	Conventional excitation	2942	45.56	0.301	0.7
	Impedance-adaptive excitation	3112	46.12	0.303	44.8
190	Conventional excitation	2795	44.10	0.321	1.6
	Impedance-adaptive excitation	2986	45.00	0.323	51.2
210	Conventional excitation	2649	42.67	0.337	1.0
	Impedance-adaptive excitation	2875	43.83	0.339	53.2

## Data Availability

The data presented in this study are available from the corresponding author upon reasonable request.
